# Hydroxyapatite-Polysaccharide Composites Synthesized from Maize Lime-Cooking Wastewater for Bone Tissue Engineering

**DOI:** 10.3390/jfb17070322

**Published:** 2026-07-04

**Authors:** Arizbe Zayas-Olivares, Mariana Franco-Morgado, Maria del Refugio Rocha-Pizaña, Wendy Ortega-Lara, Luis Martín Marín-Obispo, Janet A. Gutiérrez-Uribe

**Affiliations:** 1 Tecnologico de Monterrey, Escuela de Ingenieria y Ciencias, Av. Eugenio Garza Sada 2501 Sur, Monterrey 64849, Mexico; a01730933@tec.mx (A.Z.-O.); mariana.franco@tec.mx (M.F.-M.); mrochap@tec.mx (M.d.R.R.-P.); wlortega@tec.mx (W.O.-L.); 2 Tecnologico de Monterrey, Institute for Obesity Research, Av. Eugenio Garza Sada 2501 Sur, Monterrey 64849, Mexico; marin.martin@tec.mx

**Keywords:** hydroxyapatite, maize lime-cooking wastewater, polysaccharides, cell viability, circular economy

## Abstract

Hydroxyapatite (HAp) is a widely used bioceramic in bone tissue engineering due to its biocompatibility and osteoinductive capacity; however, sustainable low-cost synthesis routes remain a challenge. This study evaluated HAp-polysaccharide composite synthesis from nejayote, the alkaline wastewater of maize nixtamalization, via chemical precipitation with (NH_4_)_3_PO_4_ under controlled and uncontrolled pH, followed by calcination at 550 °C for 2 or 4 h. Controlled pH synthesis yielded higher solid recovery (89.8% vs. 76.4%), better calcium removal (99.8% vs. 87.4%), and smaller particle sizes (423.6 nm vs. 715.0 nm). XRD and FTIR confirmed HAp formation in both conditions, with crystallinity increasing upon calcination. Monomeric composition analysis revealed co-precipitation of amylose and arabinoxylan-derived polysaccharides in uncalcined samples, progressively eliminated by thermal treatment. Cell viability assays with human fetal osteoblasts (hFOB 1.19) confirmed non-cytotoxicity at all concentrations tested (10–633 μg/mL). Uncalcined composites synthesized without pH control achieved 126% cell viability at 633 μg/mL, surpassing pH-controlled and calcined counterparts (90–100%), suggesting active promotion of osteoblast proliferation, further supported by fluorescence imaging. These results establish nejayote as a viable dual source of calcium and polysaccharides for sustainable HAp composite synthesis with biomedical potential.

## 1. Introduction

Synthetic hydroxyapatite (HAp, general formula Ca_10_(PO_4_)_6_(OH)_2_) is the most widely used calcium phosphate bioceramic that mimics natural bone composition in terms of chemical composition, crystallographic structure, and biological behavior [1]. Its biocompatibility, non-toxicity, and osteoinductive capacity have enabled its applications in implant integration, scaffold fabrication, surface coatings, and drug delivery systems [2]. However, the large-scale use of HAp has been limited by challenges in its synthesis that require high temperatures, complex chemical reactions, and expensive materials [3]. Recently, research efforts have shifted toward more sustainable and cost-effective synthesis approaches [4]. Using controlled precipitation with (NH_4_)_3_PO_4_ as the phosphate source, high-purity HAp can be synthesized from maize lime-cooking wastewater generated during the nixtamalization process, commonly known as nejayote [5]. Beyond its calcium content for HAp production, nejayote contains a fraction of structural polysaccharides. These are primarily hemicelluloses, such as arabinoxylans, associated with the fibrous fraction (22.8–25.5%); the remaining carbohydrates consist of soluble sugars and low-molecular-weight compounds, such as free glucose, soluble starch fractions, and phenolic compounds [6]. Despite their relatively low abundance, these hemicellulosic polysaccharides exhibit valuable functional properties as stabilizers and gel-forming agents [7].

The morphology of HAp obtained through chemical precipitation can be controlled by pH or calcination [8]. pH values between 9 and 11 increased HAp solubility, allowing it to become the predominant mineral after phosphate addition and reach high saturation levels [9]. HAp synthesis has also been reported at lower pH values (3–6) with acceptable precipitation and crystal formation [10]. The pH of the synthesis medium strongly influences both HAp crystallinity and the formation of competing calcium phosphate phases. Near-neutral pH conditions reduce OH^−^ availability, favoring metastable precursor phases such as beta-tricalcium phosphate (β-TCP) and whitlockite. These phases typically form as intermediates during amorphous calcium phosphate maturation under suboptimal alkaline conditions and may persist or become dominant upon thermal treatment as previously reported for waste-derived calcium sources [11].

Additionally, calcination temperature plays a crucial role in determining crystallinity and phase purity. Lower temperatures (350 °C) produce mixed amorphous-crystalline phases with broader XRD peaks, while higher temperatures (550 °C) yield sharper, more crystalline structures [12]. However, uncalcined HAp has also been explored as a viable alternative for biomedical applications, as it retains surface-associated organic components that may enhance bioactivity and cell–material interactions [13].

Although HAp demonstrates excellent biocompatibility, it faces mechanical limitations, including low fracture toughness, susceptibility to fatigue, and insufficient mechanical strength [14]. These drawbacks can be mitigated by incorporating polymers or reinforcing agents such as polysaccharides or gelatin [15]. HAp-polysaccharide composites have emerged as particularly promising biomaterials for bone tissue engineering [16]. In this sense, arabinoxylan-HAp composites exhibit compressive strength improvements from 4.05 to 7.94 MPa [17]. Similarly, HAp-alginate nanocomposites demonstrated 79.3% and 158.4% increases in tensile strength and elastic modulus, respectively [18]. These advantages make them suitable for 3D scaffold fabrication and promising alternatives to conventional materials [19].

Nejayote presents a unique opportunity as a dual-source material for sustainable composite synthesis. This integrated approach enables the synthesis of HAp-polysaccharide composites entirely from a single waste stream. Therefore, this work synthesized HAp-polysaccharide composites from nejayote via chemical precipitation. Synthesis parameters, including pH and calcination time, were tested to evaluate the differences in cell viability and to promote the in vitro growth of osteoblasts.

## 2. Materials and Methods

### 2.1. Hydroxyapatite Synthesis

Nejayote was obtained from a local tortilla bakery in Puebla, Mexico. Samples were collected in 2 L plastic containers and stored at 4 °C until use for HAp synthesis. The nejayote exhibited a strongly alkaline pH (12.2) and the following physicochemical characteristics: COD (26.5 g/L), total solids (4.1 g/L), Ca^2+^ (1.5 g/L), SO_4_^2−^ (235.1 mg/L), PO_4_^3−^ (2.5 mg/L), total nitrogen (TN, 275 mg/L), NH_3_ (13 mg/L), NO_3_^−^ (9 mg/L), and NO_2_^−^ (0.1 mg/L).

The chemical precipitation method previously reported by Valenzuela et al., 2025 [12] was employed to synthesize HAp. 2 L of nejayote were placed into a beaker under constant stirring. Then, a concentrated solution of (NH_4_)_3_PO_4_ (98% Sigma Aldrich, St. Louis, MO, USA) was added dropwise (50 mL at 3 mL/min) at 800 RPM. The phosphate concentration was calculated to ensure that the PO_4_^3−^ ion was in excess during the reaction, according to Equation (1), where 1.42 g of PO_4_^3−^ reacts with 1 g of Ca^2+^ to form 2.5 g of HAp. Therefore, a mass ratio of 2.23 g PO_4_^3−^/g Ca^2+^ was established in the final reaction volume for the (NH_4_)_3_PO_4_ reaction, considering a dissolved Ca^2+^ concentration in the nejayote of 1.5 g/L. The starting pH of the raw nejayote was 12.31 ± 0.1.

(1) $${10Ca(OH)}_{2}+{6({NH}_{4})}_{3}{PO}_{4}\to {Ca}_{10}{\left({PO}_{4}\right)}_{6}{\left(OH\right)}_{2}+{{12NH}_{3}}^{+}+{18H}_{2}O$$

During the precipitation reaction, pH was continuously controlled using 2 M NaOH (>96% Sigma-Aldrich, St. Louis, MO, USA) to maintain a pH around 11. For the condition without pH control, no reagents were added to adjust or stabilize the pH. Once all the concentrated phosphate solution of (NH_4_)_3_PO_4_ was mixed with the nejayote, the reaction media was left under constant stirring at 800 rpm at room temperature (25 °C) for 24 h, in a process called mineral aging. The precipitate produced was recovered and cleaned through three centrifugation-resuspension cycles. To this end, the reaction medium was centrifuged (CRM Globe, Mexico City, Mexico) at 4500 rpm for 7 min, and the resulting supernatant was collected.

Ca^2+^ concentration in the supernatant was determined by inductively coupled plasma mass spectrometry (ICP-MS), using an Agilent Technologies 7800 instrument (Agilent Technologies, Santa Clara, CA, USA), monitoring the ^44^Ca isotope. Ca^2+^ removal efficiency was calculated according to Equation (2).

(2) $${Ca}^{2+}\;removal\;(\%)=\frac{{\left[{Ca}^{2+}\right]}_{nejayote}-{\left[{Ca}^{2+}\right]}_{supernatant}}{{\left[{Ca}^{2+}\right]}_{nejayote}}\times 100$$

Then, a mix of absolute ethanol (ACS grade J.T. Baker, Phillipsburg, NJ, USA) and deionized water (1:1) was used to resuspend the precipitate. The suspension obtained was centrifuged (CRM Globe, Mexico) at 4500 rpm for 7 min, and the supernatant was discarded. After that, the clean precipitates were manually collected and dried in a stove (MERMMET, Schwabach, Germany) at 105 °C for 12 h. Subsequently, the dried precipitate was calcined in a furnace (FELISA, Zapopan, Jalisco, Mexico) for 2 or 4 h at 550 °C. The calcination process yield was determined as the ratio between the mass of calcined solids and the initial mass of precipitated solids recovered from nejayote (both expressed in g), according to Equation (3). After this process, the calcined precipitates were manually milled in a mortar until the finest possible powder was obtained.

(3) $$Calcination\;process\;yield\left(\%\right)=\frac{Mass\;of\;calcined\;solids\;(g)}{Initial\;mass\;of\;precipitated\;solids\;(g)}\times 100$$

Experimental control was synthesized under the same conditions mentioned above, using CaCl_2_ as a pure calcium source (PCS) (75 mM, >96% Sigma-Aldrich, St. Louis, MO, USA): 50 mL of (NH_4_)_3_PO_4_ was dropped in 500 mL of CaCl_2_. 4.16 g of CaCl_2_ was selected to equal the concentration of dissolved Ca^2+^ detected in the nejayote. After washing and drying, the synthesized control powders were calcinated at 550 °C and then milled in a mortar as previously described.

### 2.2. Characterization Techniques

Functional groups such as phosphate (PO_4_^3−^), hydroxyl (^-^OH), and carbonate (CO_3_^2−^), related to HAp in the powders obtained after synthesis, were analyzed using a Bruker^®^ Vertex 70 FTIR spectrometer (Germany) (4000–400 cm^−1^) in ATR mode.

Particle size determination analyses by dispersive light scattering (DLS) were carried out in a Zetasizer Ultra (Malvern Panalytical, Malvern, UK) after sonicating ~5 mg of HAp powders in 1 mL of distilled water for 3 min. The dispersed samples were analyzed at a 90-degree scattering angle.

The crystalline phases present in the powders obtained were determined by X-ray diffraction (XRD) on a PANalytical Empyrean diffractometer (PANalytical, Almelo, The Netherlands) equipped with a copper anode. The patterns obtained were compared against data from the International Center of Diffraction Data (ICDD) Powder Diffraction File (PDF) database, which contains standard diffraction patterns for known crystalline phases, allowing their unambiguous identification.

The monomeric composition of the polysaccharides was analyzed by prior hydrolysis using trifluoroacetic acid (TFA) method previously reported by Muñoz-Almagro et al., 2025 [20]. For this purpose, about 30 mg of dried samples were mixed with 1.5 mL of TFA 2 M, and a stream of nitrogen was passed through and kept closed in an oven at 110 °C for 4 h. For the GC-FID analysis, 300 μL of the hydrolysate was evaporated to dryness at 40 °C using a rotary evaporator. After complete removal of TFA, 0.2 mg of phenyl-β-D-glucoside was added as internal standard (I.S.), followed by re-evaporation to dryness. The resulting monosaccharides were derivatized into their corresponding trimethylsilyl oximes derivatives as previously described [19]. Analysis was performed using an Agilent Technologies 8860 gas chromatograph coupled to a flame ionization detector (Agilent Technologies, Santa Clara, CA, USA) and equipped with a TG-5HT capillary column (30 m × 0.25 mm × 0.10 μm; Thermo Scientific TraceGold, Waltham, MA, USA) with helium as the carrier gas at a flow rate of 1 mL/min.

### 2.3. Cellular Viability Tests

HAp powders were sterilized by autoclaving (120 °C, 20 min) and were prepared according to ISO 10993-5 [21], with minor modifications, as previously described by Valenzuela et al. (2025) [12]. 60 mg of each powder was resuspended in 600 μL of culture medium (DMEM/F12 without phenol red and 100 μg/mL Antibiotic-Antimycotic BIOWEST), supplemented with 2% of fetal bovine serum (FBS), to obtain a final concentration of 0.1 g/mL. Suspensions were incubated at 37 °C (in a humidified atmosphere with CO_2_ 5%) for 24 h, then centrifuged at 8000 rpm for 2 min to obtain clear extracts.

Cell viability was assessed using the human fetal osteoblast cell line (hFOB 1.19) from the American Type Culture Collection. The cells were grown in a complete culture medium, supplemented with 10% FBS, and maintained at 37 °C (humidified atmosphere, 5% CO_2_). The hFOB 1.19 cells were seeded in 96-well plates, 2.5 × 10^4^ cells/mL (100 μL per well) and incubated overnight at 34 °C. Culture medium was subsequently replaced with 100 μL of HAp extract at different concentrations for control pH, uncontrolled pH (10, 20, 40, 79, 158, 316 and 633 μg/mL) and the complete culture medium supplemented with 10% of FBS was used as a control. After 24 h of incubation, cells were treated with 20 μL/well CellTiter 96^®^ AQueous One Solution Cell Proliferation Assay (MTS, Promega), incubated for 1 h, and the absorbance at 490 nm was measured with a 96-well plate reader (BioTek, Winooski, VT, USA).

### 2.4. Staining of Live/Dead Cells

Live cell staining was performed on hFOB 1.19 osteoblasts exposed for 24 h to nejayote extracts obtained under pH-controlled and uncontrolled conditions at a concentration of 633 μg/mL. Staining was carried out using a Live/Dead Cell Imaging Kit (Invitrogen, Waltham, MA, USA), based on calcein-AM for live cell detection and propidium iodide for dead cell identification. A 2X working solution was prepared according to the manufacturer’s instructions. The solution was applied to cells seeded at a density of 1 × 10^6^ cells and incubated for 15 min at room temperature (20–25 °C). Fluorescence imaging was conducted using a Gen5 Image Prime system (BioTek Instruments, Winooski, VT, USA). Fluorescence intensity of live cells was quantified by calculating the Corrected Cellular Fluorescence Intensity (CCFI) according to Equation (4):

(4) $$CCFI=Integreted\;Density-\left(\left(Area\;of\;selected\;cell\right)\left(Mean\;flourescence\;of\;background\right)\right)$$

### 2.5. Statistical Analysis

Data were expressed as mean ± standard deviation of three experimental replicates. Treatment means were compared using Tukey’s test, with statistical significance set at *p* < 0.05. All analyses were conducted using JMP 19 software (Cary, NC, USA).

## 3. Results

### 3.1. Yield Recovery as HAp

The final pH values after HAp precipitation varied significantly between synthesis conditions. It was maintained at 11.0 ± 0.1 for the controlled pH and it reached 6.0 ± 0.4 for the uncontrolled pH synthesis conditions (Table 1). Maintaining an alkaline pH favored direct HAp crystallization by ensuring sufficient OH^−^ availability and promoting phosphate species that stabilized HAp formation [5]. In contrast, the uncontrolled pH naturally shifted toward a pH of 6 after (NH_4_)_3_PO_4_ addition [22]. Alkaline environment promoted the co-precipitation of inorganic nejayote components and sodium hydroxide used to control pH, thereby increasing their contribution to the calcination process yield (89.8 ± 0.5%). The lower calcination process yield observed when pH was not controlled was due to the mass losses related to the removal of organic matter, as it has been observed in the recovery of HAp from other food byproducts [23]. These yields were consistent and comparable with those reported for HAp synthesized from natural calcium-rich wastes, such as periwinkle snail shells (92.12%), quail eggshells (92.01%), and oyster shells (73.65%) [24].

The calcium removal efficiency results (Table 1) clearly demonstrate the critical role of pH control in achieving complete Ca^2+^ precipitation. Under controlled alkaline conditions, removal reached 99.8 ± 0.1%. Previous reports indicate that the predominance of PO_4_^3−^ ions and elevated supersaturation favor the nucleation and growth of HAp [12]. In contrast, under uncontrolled pH conditions, removal efficiency decreased to 87.4 ± 0.6% due to the reduced availability of fully deprotonated phosphate species at suboptimal pH that limited supersaturation and weakened the thermodynamic driving force for HAp precipitation [25].

There was a statistically significant effect of pH control on particle size, obtaining an average particle size of 92.0 ± 13.6 nm for the controlled pH condition and 150.0 ± 24.1 nm for the uncontrolled pH condition (Table 1). A stable alkaline pH promotes more homogeneous nucleation and limits uncontrolled crystal growth, resulting in smaller and more uniformly dispersed particles [9].

### 3.2. Characteristics of Synthesized Powders

**Figure 1:**
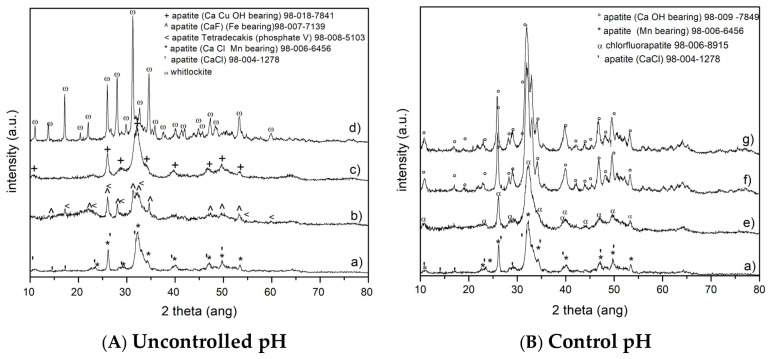
Evolution of XRD diffractograms of solids recovered after precipitation of nejayote solids using triammonium phosphate without controlled pH (panel **A**) or maintaining alkaline conditions with NaOH (Panel **B**) and compared to a control obtained using CaCl_2_ (a). Each panel also shows the differences related to the calcination process. In panel **A**: (b) uncalcined, (c) 2 h at 550 °C and (d) 4 h at 550 °C. In panel **B**: (e) uncalcined, (f) 2 h at 550 °C, and (g) 4 h at 550 °C.

In Figure 1, a thermic evolution of crystalline systems for uncontrolled and controlled samples is shown. Curves (a) in Figure 1A,B correspond to the experimental control sample, synthesized under the same conditions described above using CaCl_2_ as a pure calcium source, resulting in a dry precipitate with an apatite semicrystalline structure giving reflections indexed as apatite-type structures. In curve (b) of Figure 1A, the semicrystalline curve showed reflections from fluorcalcium apatite and a doped apatite with metals, as reported [12]. In this curve, a semicrystalline apatite-type phase was observed. Upon calcination at 550 °C, nucleation and reflection growth occur. Curve (c) of Figure 1A, with a 550 °C/2 h treatment, developed higher reflections corresponding to a crystalline apatite phase, indicating an increase in crystallinity PDF 98-018-7841. Curve (d) in Figure 1A exhibited higher intensity peaks corresponding to crystalline whitlockite (PDF 98-016-1254), a calcium phosphate phase commonly formed under near-neutral pH conditions (6.0 ± 0.4) reached during uncontrolled synthesis. The occurrence of this phase is not unique to nejayote-derived systems, as whitlockite formation after thermal treatment has also been reported for other waste-derived calcium sources, including dairy industry effluents [11] and agricultural biowastes such as eggshells and struvite [26].

In contrast, the controlled-pH system shows, in Figure 1B, the synthetic apatite (a) as a reference, and curve (e) of Figure 1B corresponded to a 2 h treatment at 550 °C and had increasing reflections to growth-crystalline levels, while curve (h) of Figure 1B showed sharper and higher-intensity peaks due to increased calcination time, as it was reported by other authors when calcination temperature increased [27]. The crystalline cell exhibits a hydroxyl apatite (PDF 98-009-7949).

The XRD patterns obtained under both synthesis conditions were consistent with mixtures of amorphous and crystalline apatite phases with possible minor ionic substitutions involving Mn, Fe, and Cu. The complex inorganic composition of nejayote, which has been reported to contain these elements (Mn: 6.59 mg/g, Fe: 277.63 mg/g, Cu: 12.87 mg/g) as determined by ICP analysis [28].

**Figure 2:**
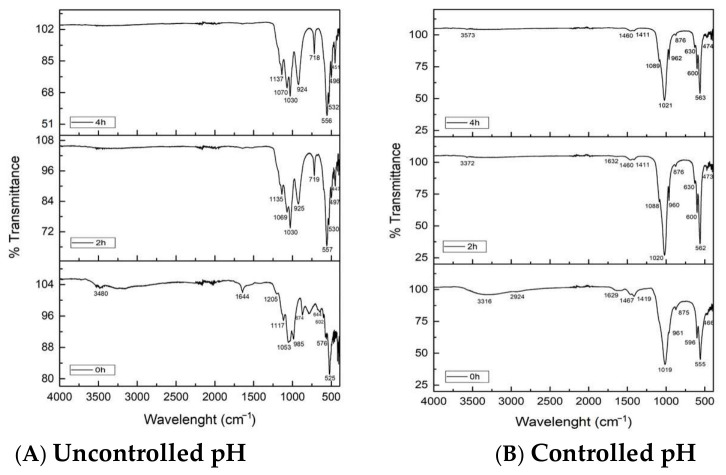
Changes in FTIR spectra of precipitates obtained: (**A**) uncontrolled pH or (**B**) control pH at different calcination times at 550 °C (uncalcinated, 2 h and 4 h).

The FTIR spectra (Figure 2) confirmed the formation of HAp in samples synthesized under controlled and uncontrolled pH conditions. Characteristic phosphate vibrations were identified at 1019–1021 cm^−1^, corresponding to asymmetric P–O stretching. A band at 960–962 cm^−1^ was assigned to symmetric PO_4_^3−^ stretching. Additionally, O–P–O bending vibrations were observed between 600 and 630 cm^−1^. These bands were consistent with previously reported HAp syntheses [12,29].

Nejayote-derived polysaccharides were evident in the samples obtained without pH control (Figure 2A) at 0 h of calcination. The broad O–H stretching band observed between 3316 and 3480 cm^−1^ is characteristic of hydroxyl-rich polysaccharide chains. The bands at 2924 and 718 cm^−1^ are associated with C–H stretching and out-of-plane bending vibrations, respectively, related to the aliphatic carbohydrate backbone of polysaccharides [30]. The spectral region associated with C–O–C glycosidic linkages (1117–1137 cm^−1^) also suggested enhanced organic–inorganic interfacial interactions during the precipitation process [31]. The band observed at 924 cm^−1^ is assigned to HPO_4_^2−^ acid phosphate vibrations, characteristic of nonstoichiometric or poorly crystalline apatite phases [32]. Additionally, the band observed at 1629–1644 cm^−1^ corresponded to C=O stretching vibrations or bound water molecules associated with the polysaccharide matrix [33].

The progressive disappearance of these bands with increasing calcination time (2 h or 4 h) confirmed the effective thermal elimination of superficially adsorbed polysaccharides, while samples with controlled pH (Figure 2B) showed cleaner spectra with predominance of the characteristic phosphate bands of HAp.

### 3.3. Monomeric Composition

In both synthesis conditions, glucose, xylose, and arabinose were the dominant monosaccharides in the uncalcined samples, confirming the incorporation of remnant maize polysaccharides in HAp precipitates obtained from nejayote (Table 2). At the uncontrolled pH condition, a higher total monosaccharide content (612.8 ± 23.2 mg/g) was obtained in comparison with the controlled pH system (499.0 ± 5.6 mg/g). Based on the monosaccharide composition, the organic fraction is mainly derived from hemicellulose and starch, which are common in corn-processing residues [34]. Starch exhibits increased solubility and swelling under alkaline conditions due to the ionization of hydroxyl groups and the disruption of granule-associated components [35]. The presence of xylose and arabinose was particularly indicative of arabinoxylan-type hemicelluloses [36]. The higher glucose content under uncontrolled pH conditions (189.9 ± 2.2 mg/g with control pH and 302.2 ± 12.2 mg/g uncontrolled pH) suggested that stronger alkalinity enhanced granule disruption and amylose solubilization [37]. In contrast, calcination led to the thermal degradation of these carbohydrates.

Minor sugars such as rhamnose, mannose, and galactose indicate the presence of more complex heteropolysaccharide structures within the nejayote-derived organic matrix. This was consistent with the reported composition of nejayote, which is rich in carbohydrates (37.8–55.7%), including structural polysaccharides such as cellulose, hemicellulose, and starch-derived fractions, as well as soluble sugars released during nixtamalization, reflecting the partial hydrolysis and leaching of maize cell wall components into the liquid phase [6].

Calcination reflects a pronounced effect on the organic fraction of the synthesized materials. The progressive thermal decomposition of polysaccharides was reflected in a marked reduction in total monosaccharide concentrations, declining to 2.4 or 3.4 mg/g after 2 h and to 1.6 or 2.5 mg/g after 4 h of thermal treatment under both synthesis conditions. These findings confirm that calcination effectively eliminates most organic components, yielding a predominantly inorganic HAp phase [38].

Galactose persisted at trace levels after 4 h of calcination in both conditions (Table 2). This anomalous thermal resistance may be attributed to galactose-containing residues such as arabinogalactans and related plant-derived glycans, whose hydroxyl groups facilitate coordination with calcium ions, promoting stronger adsorption onto the calcium phosphate matrix [39,40]. As a result, minor carbohydrate fragments may remain occluded within crystal aggregates or bound to mineral surfaces, appearing as residual monosaccharides following acid hydrolysis.

This behavior is consistent with organic–mineral interactions extensively documented in biomineralization systems, wherein polysaccharides and glycoproteins regulate calcium phosphate nucleation and crystal growth through ionic coordination and surface adsorption [41]. Under uncontrolled pH conditions, fluctuations in local supersaturation promote heterogeneous nucleation and preferential formation of amorphous calcium phosphate phases, which exhibit greater surface area and enhanced capacity for organic macromolecule occlusion.

### 3.4. Cellular Viability

**Figure 3:**
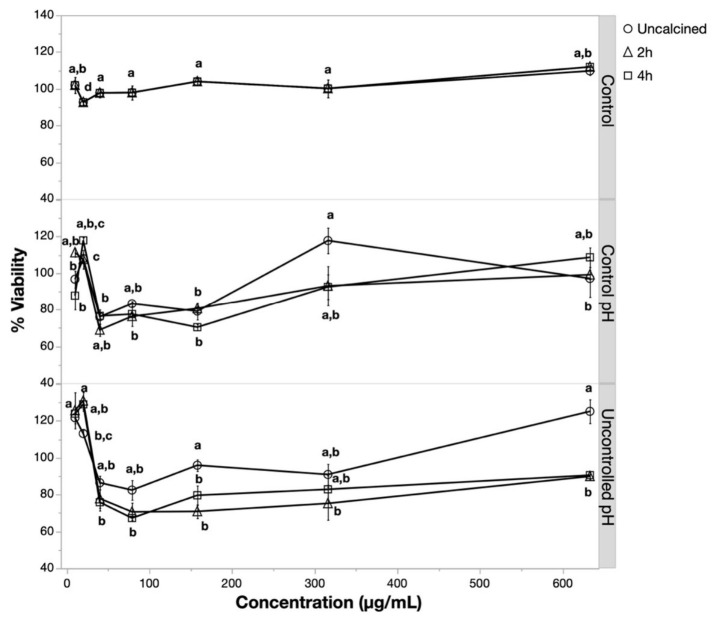
Cellular viability of human fetal osteoblast cell line performed with HAp synthesized with (NH4)_3_PO_4_ under uncontrolled and controlled pH conditions, and CaCl_2_ under controlled pH, uncalcined and calcined at 550 °C for 2 and 4 h. ^a–d^ Different letters at each tested concentration indicate significant difference (*p* < 0.05).

Osteoblasts exposed to HAp powders did not have a statistically significant negative effect on cell viability when tested at concentrations higher than 158 μg/mL (Figure 3). In fact, at 633 μg/mL, there was an increase in cell viability (126%) when uncalcined HAp obtained without pH control was compared with that obtained with controlled pH or those calcined for 2 or 4 h (90–100% viability). This not only confirmed the very low cytotoxic nature of the synthesized materials according to ISO 10993-5 guidelines for mineral particles but also the potential of these materials to promote growth. These findings were consistent with previous studies reporting that HAp-based materials preserve osteoblast viability above the 70% threshold over a broad concentration range [42]. The growth-promoting potential of HAp was not observed at 158 μg/mL, since a significant reduction in cell viability was observed in the particles obtained from nejayote with or without controlling pH when compared to controls. A similar trend was observed when the particles were tested at 79 μg/mL, reaching a cell viability down to 67% for 4 h calcined HAp obtained without pH control. These same particles at 40 μg/mL resulted in a cell viability of 76%, but 129% at 20 μg/mL or 124% at 10 μg/mL, confirming their potential to promote osteoblast growth. The moderate viability decrease at intermediate concentrations (40–158 μg/mL) was attributed to changes in the ionic composition of the culture medium. Calcium phosphate materials can alter Ca^2+^ and HPO_4_^2−^ ion concentrations in the surrounding environment in vitro, potentially affecting cell adhesion and transiently reducing viability without indicating true cytotoxicity [43].

Regarding calcination, HAp treated at 550 °C for 2 h and 4 h maintained osteoblast viability comparable to uncalcined samples. This observation is notable given that previous studies have reported variable biological responses depending on sintering temperature. Previous reports indicated that HAp sintered at relatively low temperatures (<900 °C) had increased ionic reactivity and surface area, potentially enhancing ion exchange with the culture medium and affecting in vitro biocompatibility [44]. In the present case, calcination at 550 °C for controlled times (2 and 4 h) stabilizes the material without inducing excessive ionic reactivity. Supporting this, other authors reported that uncalcined HAp had higher bioresorption and biomineralization due to its larger surface area, while calcined HAp provided a more controlled ion release profile and remains non-cytotoxic [13].

The stimulatory effect of HAp on osteoblast proliferation rather than toxicity was similar to that reported in osteoblast cells exposed to PMMA-HAp scaffolds at optimal concentrations [45]. Calcination combined with ionic substitution in nanoHAp improved cytocompatibility and supported cell proliferation, reinforcing that the thermal treatment of HAp can be tailored to enhance biological performance [46].

Overall, these results confirm that HAp synthesized under the evaluated conditions was biocompatible with osteoblasts across a clinically relevant concentration range, supporting its potential application as a bone substitute or as a coating material in biomedical devices. However, the pH of the immersion media was not measured in the present study, as calcium phosphate materials can locally alter Ca^2+^ and HPO_4_^2−^ ion concentrations and induce transient pH shifts that may influence cell viability independently of material cytotoxicity [42].

### 3.5. Effect of Hydroxyapatite (Hap) Powders on the Distribution of Live and Dead Osteoblasts

**Figure 4:**
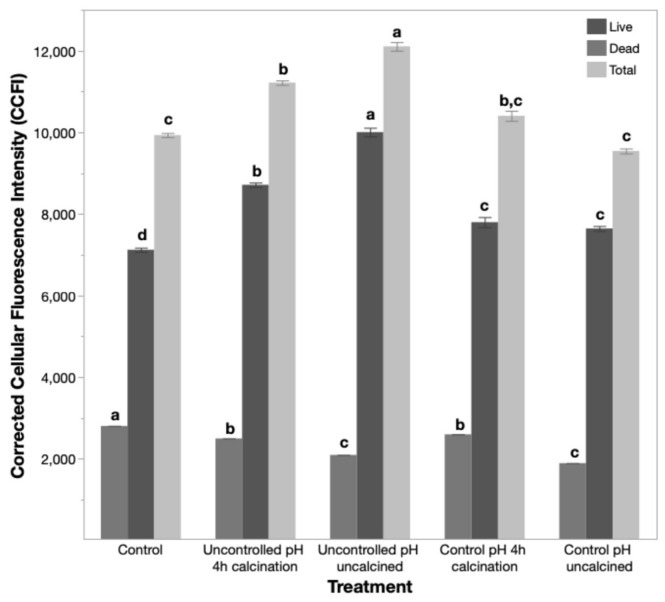
Corrected Cellular Fluorescence Intensity (CCFI) of human fetal osteoblasts (hFOB 1.19) exposed to 633 μg/mL of HAp synthesized with (NH_4_)_3_PO_4_ under uncontrolled and controlled pH conditions, uncalcined and calcined at 550 °C for 4 h, and CaCl_2_ under controlled pH. ^a–d^ Different letters at each tested concentration indicate significant difference (*p* < 0.05).

The corrected cellular fluorescence intensity (CCFI) values obtained from osteoblast cultures exposed to HAp powders at a concentration of 633 μg/mL revealed statistically significant differences in the live, dead, and total fluorescence channels (Figure 4).

The uncalcined material synthesized under uncontrolled pH conditions exhibited the highest live CCFI of 10,000, confirming the growth-promoting effect of the residual organic fraction coming from maize polysaccharides released in nejayote. Notably, this treatment also exhibited the lowest dead CCFI of 1900, indicating that the organic surface fraction not only enhanced proliferative response but also reduced membrane stress and apoptotic signaling. Consequently, this treatment yielded the highest total CCFI (12,000), reflecting the greatest overall cellular biomass per image field. The retention of these organic moieties on the HAp surface was expected to enhance material bioactivity through multiple mechanisms, including increasing surface hydrophilicity, exposing reactive functional groups such as hydroxyl and carboxyl moieties and facilitating selective protein adsorption from the culture medium [47,48]. Collectively, these surface properties are known to promote osteoblast adhesion, spreading, and subsequent intercellular interactions that drive the formation of cellular aggregates [49]. However, it is important to note that the surface-associated polysaccharide fraction retained in uncalcined materials may serve as a substrate for microbial growth, representing a sterility challenge for long-term storage and clinical use. In HAp-polysaccharide composite systems, UV exposure has been reported as an effective sterilization strategy that preserves the integrity of the organic fraction [50].

Calcination of this material (4 h) significantly reduced live CCFI to 8700, while dead CCFI increased to 2600. This shift likely diminished the availability of bioactive surface functionalities, as organic fractions and surface complexity are known to enhance cell–material interactions and osteoblast response [51]. Consequently, fewer adhesion sites were available, limiting cell attachment and clustering, despite the increase in crystallinity associated with calcination [45]. The total CCFI of this treatment (11,000) remained significantly higher than that of the control, confirming that even after calcination, uncontrolled pH-derived HAp retains meaningful bioactivity. This sustained bioactivity may also be partially attributed to the presence of whitlockite, the dominant crystalline phase identified by XRD in calcined uncontrolled pH samples, which has been reported to promote osteoblast proliferation, cell adhesion, and osteogenic differentiation [52].

Conversely, HAp particles obtained under controlled pH did not show a statistically significant difference before and after 4 h of calcination in the live CCFI (7900 and 7800, respectively) and dead CCFI values (2500 to 2100). The thermal stability of bioactivity can be attributed to the well-defined stoichiometric composition achieved under controlled pH conditions. The synthesis method and pH significantly affect the phase composition, particle size, and Ca/P ratio of HAp, which directly influence solubility and bioactivity [53]. Furthermore, controlled pH synthesis likely yields more homogeneous and less agglomerated particles, allowing the surface interaction profile to remain largely unaffected after calcination [54]. Notably, both groups exhibited total CCFI values significantly higher than the control, confirming that controlled pH yields HAp with superior intrinsic surface bioactivity regardless of thermal treatment status.

In contrast, the control with HAp synthesized from CaCl_2_ exhibited the lowest live CCFI among all groups (7100), and the highest dead CCFI (2800), likely due to its tendency to form calcium-deficient, poorly crystalline, and non-stoichiometric phases with suboptimal Ca/P ratios [55]. The presence of structural disorder and residual ionic impurities can negatively affect surface reactivity and reduce the availability of bioactive sites for cell interaction [56]. Although it maintained a measurable cellular response above baseline, confirming intrinsic HAp biocompatibility.

Differences in total CCFI values across treatment groups reflect the differential proliferative responses produced by each HAp surface. Materials that support greater cell proliferation result in increased total cellular biomass per imaging field, which is reflected in proportionally higher combined fluorescence signals. This interpretation is supported by the viability assay results, where no treatment induced cytotoxicity exceeding acceptable thresholds, confirming that differences in total CCFI are attributable to cell density rather than cell death or loss of membrane integrity.

Representative Live/Dead fluorescence micrographs obtained at 633 μg/mL for uncalcined samples (Supplementary material Figure S1) corroborated the CCFI results. All conditions displayed predominantly green fluorescence with minimal red signal, confirming high osteoblast viability consistent with the viability of assay results. The uncontrolled pH composite (Supplementary material Figure S1A) exhibited a higher density of individually dispersed live cells with more intense and heterogeneous green fluorescence, which may reflect enhanced cell–material interactions promoted by the surface-associated polysaccharide fraction retained under these synthesis conditions [47]. In contrast, cells exposed to the controlled pH material (Supplementary material Figure S1B) displayed a more organized and aggregated morphology characteristic of osteoblast monolayer formation, suggesting a more stable and homogeneous cell–surface interaction profile, consistent with the well-defined stoichiometric composition of this material [54]. The CaCl_2_-derived HAp (Supplementary material Figure S1C) showed comparable viability but with a notably lower cell density and less defined cellular clustering, in agreement with its reduced CCFI values, likely reflecting its suboptimal surface reactivity and Ca/P stoichiometry [56].

## 4. Conclusions

This study confirms that nejayote, constitutes a viable and sustainable dual source of calcium and polysaccharides for the synthesis of HAp-polysaccharide composites with biomedical potential. pH control during chemical precipitation was identified as a critical parameter, as alkaline conditions (pH 11) favored near-complete calcium removal (99.8%), higher solid yields (89.8%), and the formation of smaller, more homogeneous particles (423.6 nm) compared to uncontrolled conditions. XRD and FTIR analyses confirmed that calcination at 550 °C progressively improved HAp crystallinity and effectively eliminated the organic fraction, yielding a predominantly inorganic mineral phase after 2 or 4 h of thermal treatment.

The monomeric composition analysis revealed that nejayote-derived hemicellulosic polysaccharides, primarily arabinoxylans and starch-related fractions, co-precipitate with HAp under both synthesis conditions, with a higher organic retention observed under uncontrolled pH. Calcination effectively degraded most polysaccharides, although trace galactose residues persisted, likely due to strong coordination interactions with the calcium phosphate matrix.

Cell viability assays in the human fetal osteoblast (hFOB 1.19) cell line confirmed the non-cytotoxic nature of all synthesized materials in accordance with ISO 10993-5 guidelines. Notably, osteoblast proliferation increased with concentration, with the most pronounced effect observed at 633 μg/mL, where uncalcined HAp synthesized without pH control achieved 126% cell viability, surpassing all other conditions. This concentration-dependent proliferative response suggests that higher particle concentrations actively stimulate osteoblast growth. Minor viability reductions at intermediate concentrations (40–158 μg/mL) were attributed to transient ionic disturbances in the culture medium rather than intrinsic cytotoxicity. Calcination at 550 °C for 2–4 h preserved biocompatibility comparably to uncalcined samples, confirming that controlled thermal treatment effectively stabilizes the material without adverse biological effects.

Furthermore, fluorescence imaging revealed that uncalcined composites synthesized under uncontrolled pH conditions exhibited the highest corrected total cell fluorescence, suggesting that the preservation of surface-associated polysaccharides enhances osteoblast adhesion and intercellular interactions. In contrast, controlled pH synthesis yielded materials with stable bioactivity independent of calcination status, attributed to their more homogeneous stoichiometric composition.

These results highlight the dual valorization potential of nejayote as both a mineral and polysaccharide source, positioning nejayote-derived HAp composites as promising functional biomaterials for bone regeneration applications within a circular bioeconomy framework. However, an important risk that must be addressed before clinical translation is the long-term sterility of uncalcined HAp-polysaccharide composites. Although sterilization by autoclaving (120 °C, 20 min) was validated prior to in vitro testing, the surface-associated polysaccharide fraction retained in uncalcined materials may serve as a substrate for microbial growth during storage, potentially compromising sterility over time. Future studies should therefore evaluate the long-term sterility stability of these composites under different storage conditions, as well as the effect of alternative sterilization methods on the integrity of the polysaccharide fraction and its associated bioactivity.

Additionally, measurement of the immersion media pH across all tested materials and concentrations is recommended to more precisely decouple the contributions of phase composition, polysaccharide content, and ionic environment to the observed biological responses. Further characterization, including scanning electron microscopy (SEM) analysis, is also warranted to better understand the morphological and interfacial properties.

## Figures and Tables

**Table 1:** Comparison of the final pH after precipitation of nejayote solids using triammonium phosphate maintaining alkaline conditions with NaOH or without controlled pH and changes in calcination process yield, calcium removal efficiency and average particle size of total solids recovered after 2 h of calcination at 550 °C.

Synthesis Condition	pH After Synthesis	Calcination Process Yield (%)	Ca^2+^ Removal Efficiency (%)	Average Particle Size (nm)
Control pH	11.0 ± 0.1 ^a^	89.8 ± 0.5 ^a^	99.8 ± 0.1 ^a^	92.0 ± 13.6 ^a^
Uncontrolled pH	6.0 ± 0.4 ^b^	76.4 ± 0.2 ^b^	87.4 ± 0.6 ^b^	150.0 ± 24.1 ^b^

^a,b^ Different letters in each column indicate statistically significant differences.

**Table 2:** Monomeric composition of polysaccharides obtained from synthesized powders of uncontrolled and control pH at different calcination times at 550 °C (uncalcined, 2 h and 4 h). Data expressed as mg/g polysaccharides. ND: not detected.

	Monosaccharide Content (mg/g)					
	Control pH			Uncontrolled pH		
Monosaccharide	Uncalcined	2 h Calcination	4 h Calcination	Uncalcined	2 h Calcination	4 h Calcination
Xylose	143.0 ± 12.2 ^a^	ND *	ND	154.4 ± 8.4 ^a^	ND	ND
Arabinose	103.9 ± 15.0 ^a^	ND	ND	104.6 ± 0.2 ^a^	ND	ND
Rhamnose	UQL ^#^	ND	ND	UQL	ND	ND
Galactose	59.0 ± 0.9 ^a^	UQL	UQL	45.3 ± 1.8 ^b^	UQL	UQL
Mannose	UQL	UQL	ND	UQL	ND	ND
Glucose	189.9 ± 2.2 ^b^	UQL	UQL	302.2 ± 12.2 ^a^	UQL	UQL
Totals	499 ± 5.6 ^b^	UQL	UQL	612.8 ± 23.2 ^a^	UQL	UQL

^a,b^ Different letters in each row indicate statistically significant differences. * ND = no peak was detected. ^#^ UQL = a peak was detected but under quantification lower limit.

## Data Availability

The original contributions presented in this study are included in the article/Supplementary material. Further inquiries can be directed to the corresponding author.

## Supplementary Materials

The following supporting information can be downloaded at: https://www.mdpi.com/article/10.3390/jfb17070322/s1, Figure S1. Representative micrographs of live/dead fluorescence of human fetal osteoblasts (hFOB 1.19) after 24 h exposure to uncalcined HAp at 633 μg/mL synthesized under: (A) uncontrolled pH, (B) controlled pH and (C) control CaCl_2_. Scale bar = 300 μm. Scale bar = 300 μm.
