# Supplementary material for: Hydroxyapatite-Polysaccharide Composites Synthesized from Maize Lime-Cooking Wastewater for Bone Tissue Engineering
Source: J Funct Biomater. 2026 Jul 4;17(7):322. doi: 10.3390/jfb17070322 (PMC13412145; doi:10.3390/jfb17070322)
Supplement: Supplementary file 1 [file jfb-17-00322-s001.zip › jfb-4322615-supplementary.pdf]

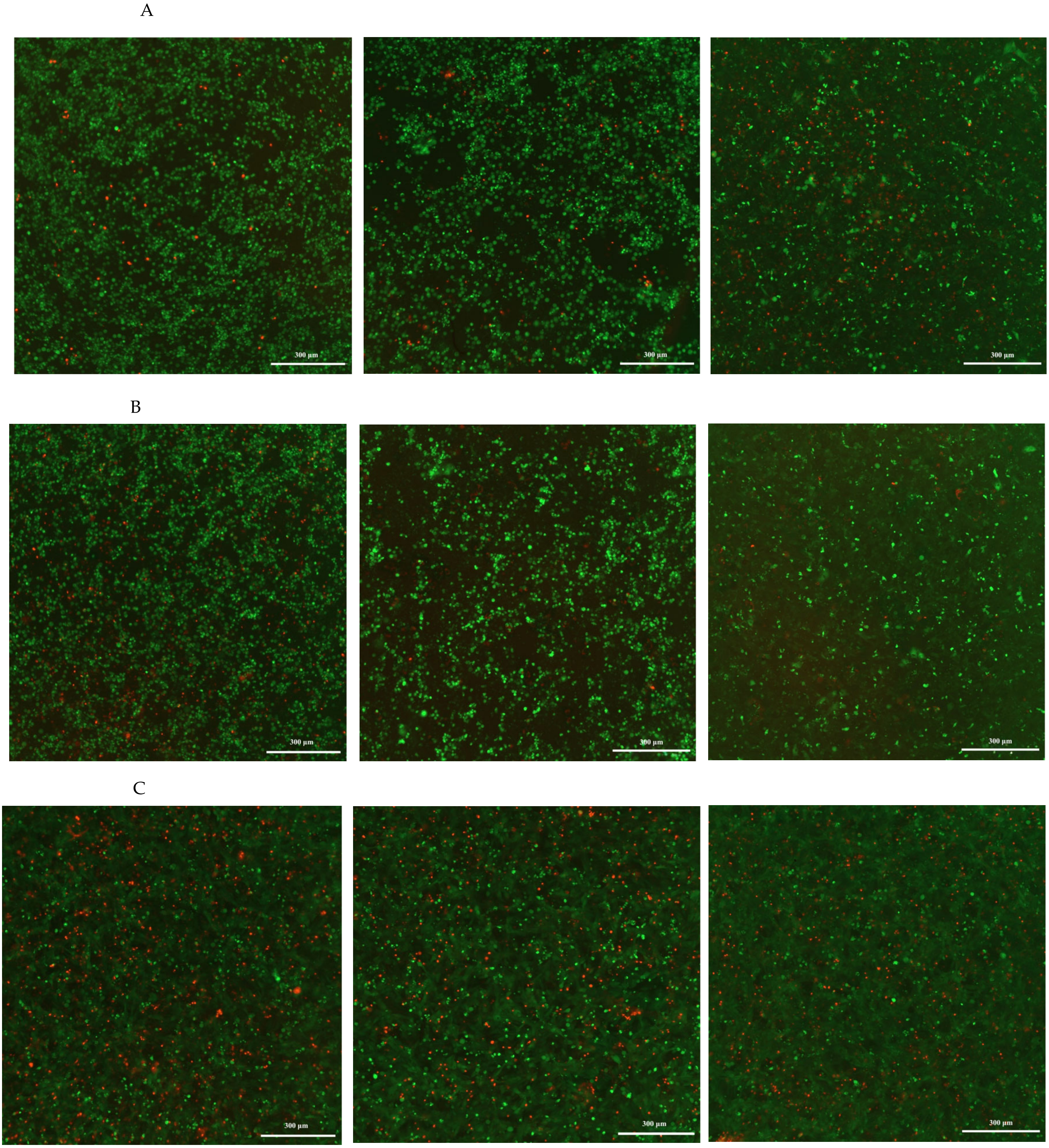
A
B
C
300 µm

Figure S1. Representative micrographs of live/dead fluorescence of human fetal osteoblasts (hFOB 1.19) after 24 h exposure to uncalcined HAp at 633 μg/mL synthesized under: (A) uncontrolled pH, (B) controlled pH and (C) control $CaCl_2$. Scale bar = 300 μm. Scale bar = 300 μm.
